# Endoscopic management of complex colorectal anastomotic leakage with a pelvic collection and rectovaginal fistula

**DOI:** 10.1055/a-2178-4008

**Published:** 2023-11-08

**Authors:** Laurent Monino, Radu Bachmann, Daniel Leonard, Christophe Remue, Etienne Danse, Alex Kartheuser, Tom Moreels

**Affiliations:** 1 70492 Department of Hepatogastroenterology, Cliniques universitaires Saint-Luc, Bruxelles, Belgium; 2 70492 Department of Colorectal Surgery, Cliniques universitaires Saint-Luc, Bruxelles, Belgium; 3 70492 Radiology Department, Cliniques universitaires Saint-Luc, Bruxelles, Belgium

The adverse event rate after colorectal surgery is up to 20%, with anastomotic leakage representing 2.9%–15.3% of events 1 . Surgical management consists of external drainage of the collection or challenging redo surgery. Endoscopic vacuum therapy (EVT) is based on the application of negative pressure on tissues in order to drain pus and favor granulation tissue 2 . The clinical success rate of EVT for the closure of collections is around 70%–80% 3 4 5 . We report the case of a patient with complex colorectal anastomotic leakage with a large presacral abscess and rectovaginal fistula, who was successfully treated with endoscopic drainage along with EVT.

A 56-year-old woman was diagnosed with rectal adenocarcinoma and underwent laparoscopic anterior resection of the rectum with lymph node dissection and colorectal anastomosis. She presented 1 month later with anastomotic leakage complicated by a presacral abscess and a rectovaginal fistula ( Fig. 1 ). The collection was initially surgically drained without success. Endoscopy was performed, with the patient under general anesthesia, with fluoroscopy and demonstrated a two-third circumferential dehiscence of the colorectal anastomosis, associated with a complex pelvic collection and a rectovaginal fistula ( Fig. 2 ). The multiloculated pelvic collection was drained by placing multiple double-pigtail stents in association with a sponge connected to an external vacuum collector ( Video 1 ). The sponge was replaced endoscopically every 3–4 days. After 2 weeks, the double-pigtail stents were removed. At day 26, the collection had resolved and the leak had successfully closed. Endoscopic and radiologic follow-up at 2 and 9 months showed a normal colorectal anastomosis with no recurrence of the pelvic collection or rectovaginal fistula ( Fig. 3 ).

**Fig. 1:**
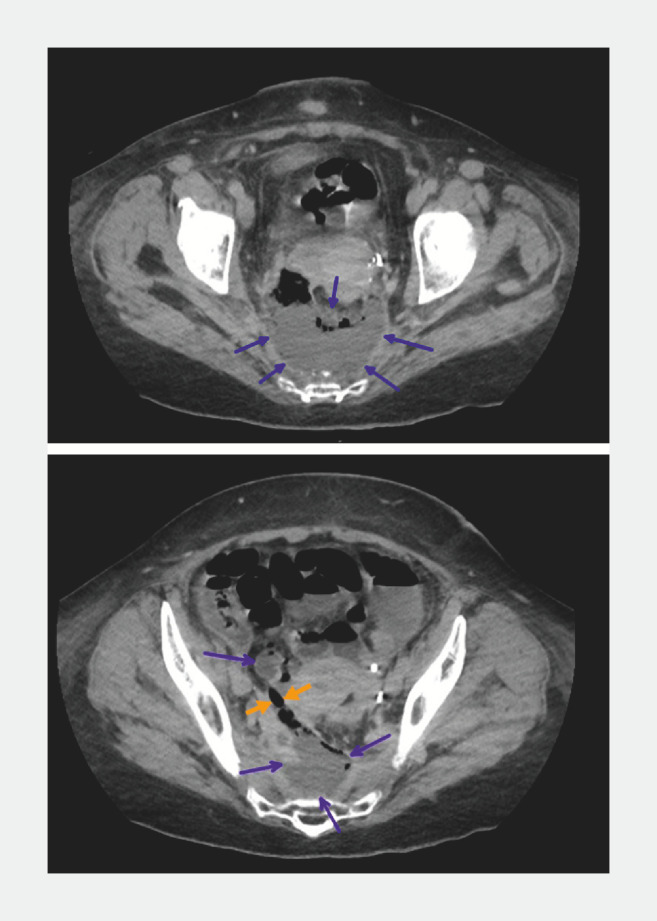
Computed tomography image before surgical drainage showing a complex pelvic collection (blue arrows) associated with a rectovaginal fistula (yellow arrows).

**Fig. 2:**
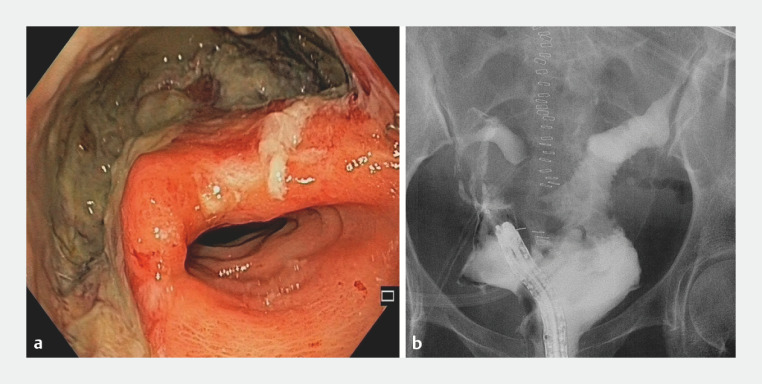
Assessment prior to treatment showing: **a** on endoscopic view, colorectal anastomotic leakage complicated by a pelvic collection; **b** on fluoroscopic view, a complex pelvic collection associated with a rectovaginal fistula.

Endoscopic management of a complex colorectal anastomotic leakage using double-pigtail stents and endoscopic vacuum therapy.Video 1

**Fig. 3:**
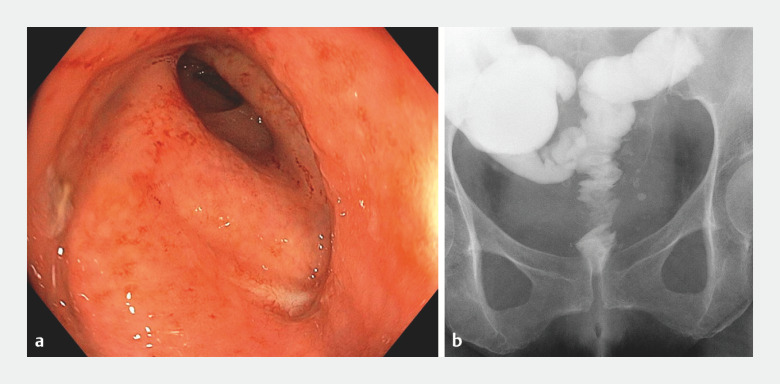
Follow-up at 9 months showing: **a** on endoscopic view, complete healing of the colorectal anastomotic leakage; **b** on fluoroscopic view, recovery of digestive tract integrity.

The management of adverse events after colorectal surgery requires a multidisciplinary approach. This combined endoscopic treatment was shown to be a good option in this patient with complex colorectal anastomotic leakage. EVT is a useful tool for the management of colorectal anastomotic leakage. Nevertheless, a prospective comparative study between endoscopic treatment and redo surgery is warranted.

Endoscopy_UCTN_Code_TTT_1AQ_2AG

## Notes

Endoscopy E-Videos https://eref.thieme.de/e-videos
*E-Videos* is an open access online section of the journal *Endoscopy* , reporting on interesting cases and new techniques in gastroenterological endoscopy. All papers include a high-quality video and are published with a Creative Commons CC-BY license. Endoscopy E-Videos qualify for HINARI discounts and waivers and eligibility is automatically checked during the submission process. We grant 100% waivers to articles whose corresponding authors are based in Group A countries and 50% waivers to those who are based in Group B countries as classified by Research4Life (see: https://www.research4life.org/access/eligibility/ ). This section has its own submission website at https://mc.manuscriptcentral.com/e-videos .
